# A Rare Case of Tuberculous Nodular Breast Abscess in an Immunocompetent Indian Female

**DOI:** 10.7759/cureus.45977

**Published:** 2023-09-26

**Authors:** Sankalp Yadav

**Affiliations:** 1 Medicine, Shri Madan Lal Khurana Chest Clinic, New Delhi, IND

**Keywords:** mycobacterium tuberculosis (mtb), cbnaat/xpert/rif assay, fine-needle aspiration cytology (fnac), tuberculosis, breast abscess

## Abstract

In underdeveloped countries, *Mycobacterium tuberculosis* infection is common and typically manifests as pulmonary tuberculosis. Nevertheless, reports of tuberculous extrapulmonary involvement are also documented. Tuberculous breast abscess is a rare presentation, especially in immunocompetent hosts. Herein, a case of primary tuberculous breast abscess is presented. The patient came with complaints of pain in her right breast and a discharging sinus. A thorough diagnostic workup resulted in the establishment of a diagnosis with the detection of *Mycobacterium tuberculosis* on smear microscopy of pus, a cartridge-based nucleic acid amplification test, a line probe assay, and culture. Ultimately, she was put on anti-tubercular treatment.

## Introduction

Tuberculosis is a serious hazard to global health [1]. *Mycobacterium tuberculosis* infection is the disease's primary cause, and it is mainly disseminated by inhaling aerosols tainted with the bacteria [2]. There are two different manifestations of tuberculosis: extrapulmonary tuberculosis, which refers to infections outside of the lungs, and pulmonary tuberculosis, when the foci of the primary infection lie in the lungs [3]. Approximately 17.5% of all cases of tuberculosis are extrapulmonary [4].

The extrapulmonary manifestations of tuberculosis in the breast are very rare [5]. This clinical condition is mostly seen in multiparous lactating females of the Indian subcontinent and Africa and makes up about 0.1% of all cases of tuberculosis manifesting at extrapulmonary sites [4,6]. This category includes about 3% of all breast conditions necessitating surgical operations [7]. Its incidence ranges from 3% to 4.5% in India [8].

The current case is an extremely uncommon type of extrapulmonary tuberculosis that manifested as a primary tuberculous unilateral breast abscess in an Indian multiparous housewife where both lung foci and a history of tuberculosis were absent. The final diagnosis was reached after a thorough clinical evaluation, and she began anti-tubercular therapy in accordance with national recommendations.

## Case presentation

A 28-year-old Indian female from a low-income family who is not diabetic complained of a painful lump within her right breast for a period of one month. She was well until one month ago when she noticed a 3 cm-sized lump in her right breast. This lump was insidious in onset and was associated with pain for 25 days. The pain was mild, continuous, non-radiating, and not linked with any aggravating or alleviating factors. Further, there has been a purulent discharge from this lump for the past five days.

There was no prior history of fever, cough, weight loss, night sweats, or lumps in the opposite breast or axilla. Additionally, there was no history of tuberculosis in her or any of her contacts. She was a housewife, and there was no history of smoking, alcoholism, or any substance abuse. Besides, there was no history of stays at night shelters, refugee camps, or imprisonment.

**Figure 1:**
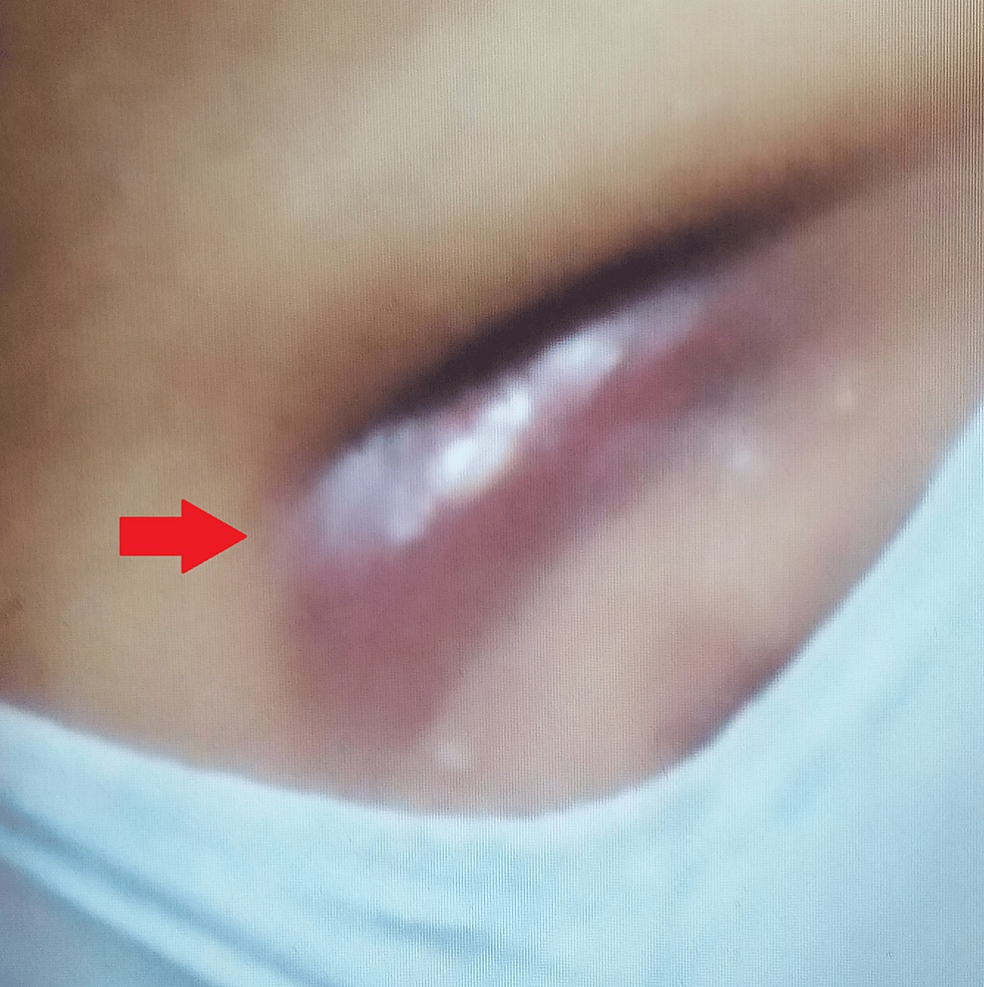
Gross image showing discharging sinus

A general examination was suggestive of a hemodynamically stable female. Local examination revealed a tender, ill-defined, firm-to-hard, irregular lump approximately 3 cm × 3 cm in size, with a discharging sinus in the right breast. There was no attachment to the skin or underlying chest wall, and the nipple was not retracting (Figure 1).

Also, there was no cyanosis; clubbing; jaundice; cervical, axillary, or supraclavicular lymphadenopathy; or pallor. Her obstetric history was as follows: gravida 2, para 2; living, 2; and abortion, 0. She was 24 when she gave birth to her first child. She breastfed each child for 13 months. Her age at menarche was 12. Moreover, her systemic examination was unremarkable.

**Figure 2:**
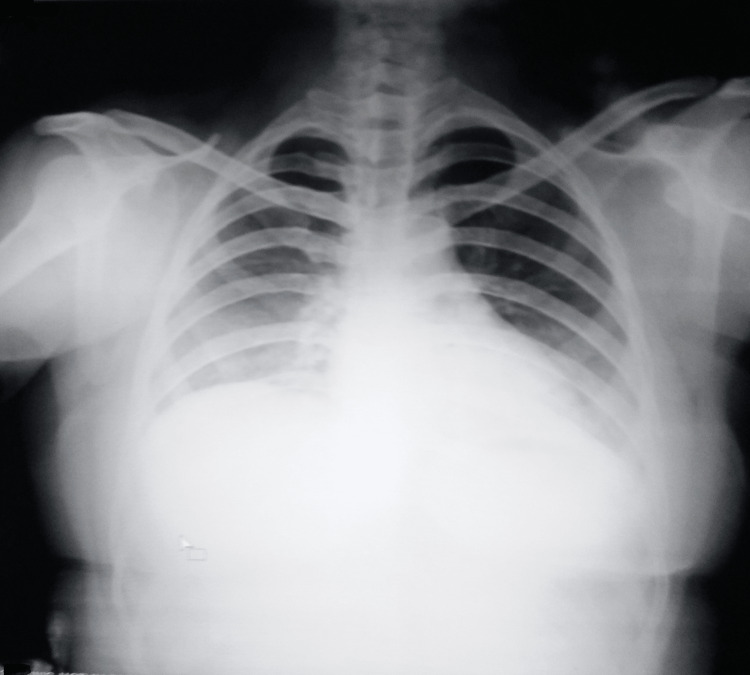
An unremarkable chest radiograph (PA view) PA: posteroanterior

A likely diagnosis of pyogenic breast abscess was made using the differentials of tuberculous breast abscess and breast cancer. She was advised routine blood tests, a chest ultrasound, and a chest radiograph. Her chest radiograph showed no abnormalities (Figure 2).

**Figure 3:**
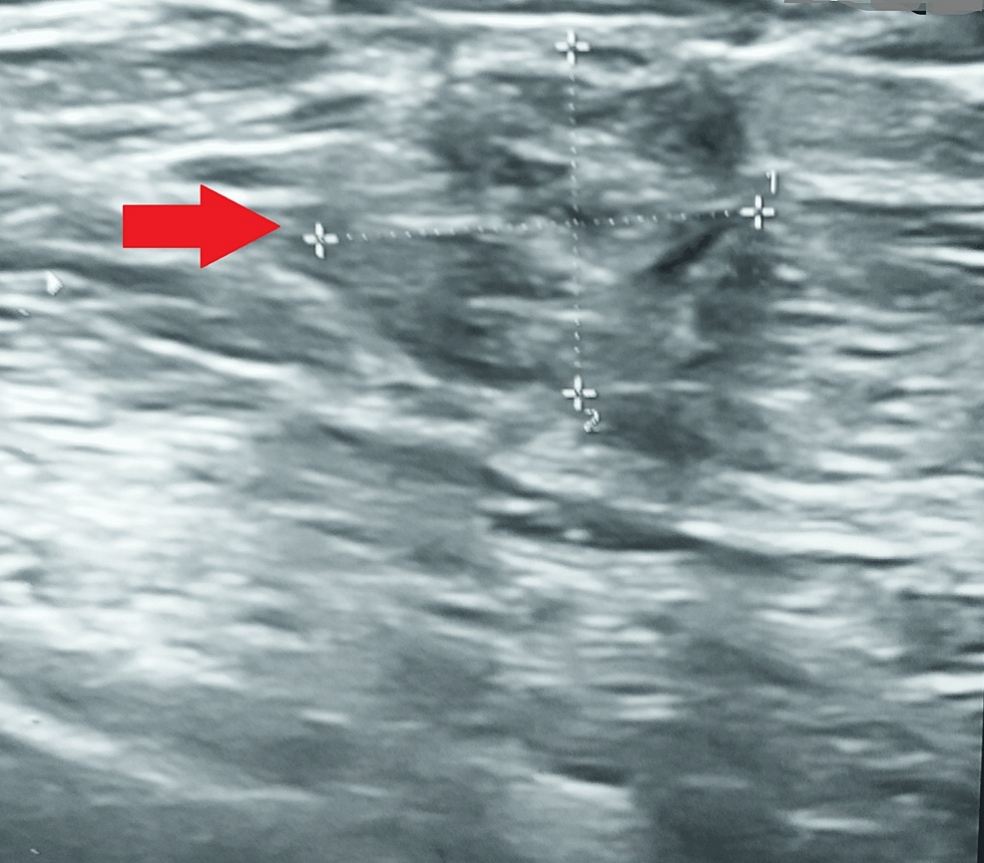
A chest ultrasound suggestive of an irregular collection

A chest ultrasound was suggestive of an irregular, heterogeneous cystic collection in the right axillary tail region (10 o'clock position) measuring about 19 × 16 × 14 (volume: approximately 3 cc) with the presence of multiple lymph nodes surrounding the lesion (with intact fatty hilum), the largest measuring 22 × 10 mm (Figure 3).

The right breast lump's fine-needle aspiration cytology was done, which resulted in the removal of 20 cc of yellow-colored pus. The fluorescent smear microscopy for acid-fast bacilli from the specimen was positive for *Mycobacterium tuberculosis*. Similar findings were reported on the cartridge-based nucleic acid amplification test (low detection of *Mycobacterium tuberculosis* without resistance to rifampicin) and the line probe assay. Another sample was sent for liquid culture and drug susceptibility testing, where there was growth of *Mycobacterium tuberculosis* with no resistance to first-line anti-tubercular drugs. Further, the incision and drainage of the abscess were done, and the histopathology of the samples was suggestive of granulomas with epitheloid cells, lymphocytes, and Langhans giant cells in a necrotic background with no evidence of malignancy. Laboratory investigations were remarkable for a raised erythrocyte sedimentation rate (76 mm in the first hour). Her human immunodeficiency viruses I and II were nonreactive, but her Mantoux test was positive.

Based on the findings, a final diagnosis of primary tuberculous abscess of the right breast was made, and she was advised to take a fixed-dose combination of anti-tubercular medicines per her weight according to the national guidelines (isoniazid, rifampicin, pyrazinamide, and ethambutol for 56 days and then isoniazid, rifampicin, and pyrazinamide for four months).

Initially, she did fine on her treatment, with no negative drug response, a reduction in her pain, and a stoppage of discharge. After two months of treatment, at her request, she was transferred to her hometown. Reports of her last follow-up were inaccessible. However, her outcome was marked as treatment complete in the national data software.

## Discussion

In endemic nations, tuberculosis significantly impacts public health [9]. Extrapulmonary tuberculosis of the breast is seldom reported [10]. It is mainly seen in females aged 20-40 years, but no age is immune [11,12]. Isolated cases in males are also available in the literature [11]. It is categorized as primary if it solely affects the breast and is absent from the rest of the body or secondary if it affects other body regions as well [12]. Compared to primary breast abscesses, secondary ones are somewhat more frequent [10,11]. Additionally, per the latest classification, breast tuberculosis could manifest as a nodular, disseminated, or tuberculous abscess [13]. The present case was of nodular type, as it presented as a distinct mass that progressively involved the skin, resulting in ulceration.

Diagnosis is challenging as mammography has low sensitivity [13]. Ultrasonography could identify about 60% of lesions [14]. Ziehl-Neelsen staining, or culture-based detection of *Mycobacterium tuberculosis*, is the gold standard for diagnosis [13]. In order to identify the bacilli, fine-needle aspiration cytology is typically used to look for stains, epitheloid cells, and granulomas [13]. The results of fine-needle aspiration cytology are normally equivocal, and even in cultures, the acid-fast bacilli are frequently undetectable [13]. The identification of the pervasive granulomatous inflammation with caseous necrosis from the lesion's histopathology, as done in this case, is very helpful [13].

Management is essentially medical, with anti-tubercular drugs [6,10]. Incision and drainage are recommended; however, surgical intervention without anti-tubercular drugs is usually futile [10,13].

In a study by Samal et al., only four out of 84 cases of breast abscess had discharging sinuses [15]. Ghalleb et al. reported 12 cases of breast tuberculosis [16]. They concluded that radiological imaging and the clinical examination were not specific, and the diagnosis required either histological confirmation or positive cultures of Koch bacillus [16].

A case of tuberculous nodular breast abscess is presented, which was timely managed. The paucity of data about this condition emphasizes the need for data from endemic countries, as large-scale studies will be helpful in increasing awareness and drafting new management guidelines for this rare clinical condition.

## Conclusions

This article presents a case of a primary tuberculous breast abscess in an Indian female who complained of pain in her right breast and a discharge from a sinus. In the absence of the constitutive symptoms of tuberculosis and a history of contact, the definitive diagnosis was made after a thorough diagnostic investigation, and she started anti-tubercular therapy. The case required a significant degree of skepticism for a timely diagnosis and care, as any delay could have yielded unfavorable results.
